# A Qualitative Study Exploring Menstruation Experiences and Practices among Adolescent Girls Living in the Nakivale Refugee Settlement, Uganda

**DOI:** 10.3390/ijerph17186613

**Published:** 2020-09-11

**Authors:** Elizabeth Kemigisha, Masna Rai, Wendo Mlahagwa, Viola N. Nyakato, Olena Ivanova

**Affiliations:** 1 Faculty of Interdisciplinary Studies, Mbarara University of Science and Technology, P.O. Box 1410 Mbarara, Uganda; ekemigisha@must.ac.ug (E.K.); mlahagwa071@yahoo.com (W.M.); vnyakato@must.ac.ug (V.N.N.); 2 Helmholtz Zentrum München, German Research Centre for Environmental Health, 85764 Neuherberg, Germany; masna.rai@helmholtz-muenchen.de; 3 Division of Infectious Diseases and Tropical Medicine, Medical Centre of the University of Munich (LMU), 80802 Munich, Germany

**Keywords:** refugee, adolescent, menstruation, sexual and reproductive health, migration, Uganda, Africa

## Abstract

(1) ***Background***: Girls in low- and lower-middle income countries face challenges in menstrual health management (MHM), which impact their health and schooling. This might be exacerbated by refugee conditions. This study aimed at describing menstruation practices and experiences of adolescent girls in Nakivale refugee settlement in Southwestern Uganda. (2) ***Methods***: We conducted a qualitative study from March to May 2018 and we intentionally selected participants to broadly represent different age groups and countries of origin. We conducted 28 semistructured interviews and two focus group discussions. Data were transcribed and translated into English. Analysis included data familiarization, manual coding, generation and refining of themes. (3) ***Results***: Main findings included: (a) challenging social context with negative experiences during migration, family separation and scarcity of resources for livelihood within the settlement; (b) unfavorable menstruation experiences, including unpreparedness for menarche and lack of knowledge, limitations in activity and leisure, pain, school absenteeism and psychosocial effects; (c) menstrual practices, including use of unsuitable alternatives for MHM and poor health-seeking behavior. (4) ***Conclusions***: A multipronged approach to MHM management is crucial, including comprehensive sexual education, enhancement of parent–adolescent communication, health sector partnership and support from NGOs to meet the tailored needs of adolescent girls.

## 1. Introduction

Uganda is one of the world’s largest refugee hosting countries and is currently home to 1,331,565 refugees [1]. Women comprise 52% of the refuge population in Uganda, with 8% of the total population being girls between 12 and 17 years old [1]. Evidence suggests that sexual and reproductive health (SRH) of women and girls might be negatively influenced by the challenges due to migration and displacement [2,3]. Available evidence demonstrates that knowledge of young refugee and displaced women and girls regarding contraceptive methods, STIs and HIV/AIDS are limited. They frequently experience gender-based and sexual violence. Distance, costs and stigma often limit their access to, and availability of, SRH services [3].

Menstruation is a crucial aspect of SRH and unmet menstrual needs impact girls’ education, health and well-being [4]. As of 2017, around 26 million menstruating girls and women were displaced due to disasters and conflicts [5]. Girls in humanitarian crisis face particular challenges related to menstruation and access to menstrual hygiene products. The World Health Organization (WHO) and the United Nations Children’s Fund (UNICEF) defined menstrual hygiene management (MHM) as:

*“Women and adolescent girls are using a clean menstrual management material to absorb or collect menstrual blood, that can be changed in privacy as often as necessary for the duration of a menstrual period, using soap and water for washing the body as required, and having access to safe and convenient facilities to dispose of used menstrual management materials. They understand the basic facts linked to the menstrual cycle and how to manage it with dignity and without discomfort or fear.”*.

[6]

There are only a handful of studies describing knowledge and experiences of refugee and displaced young women and girls around menstruation and MHM. Available studies show that there is a significant lack of materials and facilities for MHM, as well as lack of education about menstruation and SRH [7,8]. Research into the menstrual practices and experiences of refugee girls living in Uganda also remains scarce. The few studies performed on local Ugandan schoolgirls demonstrate that girls experience embarrassment and fear of teasing related to menstruation, missing school due to menstrual pain, lack of effective materials for MHM, as well as lack of information regarding menstruation and MHM [9,10,11]. This situation might be even more exacerbated for girls and young women living in a humanitarian setting in Uganda. Quantitative findings from the study performed in the Nakivale refugee settlement in Uganda showed that from 260 girls who participated in the survey, 78% had access to disposable pads, followed by 18% who used cotton cloth made from rags. A total of 43% of girls missed school during their menstruation [12].

Thus, to respond to the increased international attention on empowering girls through the Sustainable Development Goals (SDGs), more evidence is need on menstruation and MHM in humanitarian settings and the solutions to address these challenges. This qualitative study aimed to address the existing knowledge gap by describing the context and highlighting challenges faced by adolescent refugee girls during migration and their stay at the refugee settlement in Uganda. We further described menstruation practices and experiences of these adolescent refugee girls.

## 2. Materials and Methods

This qualitative study was conducted in the Nakivale refugee settlement located in Isingiro District, Southwestern Uganda, which is hosting approximately 117,894 refugees [1]. Data were collected from March to May 2018. More details about the study setting and design are provided elsewhere [12]. This cross-sectional study was based on the postpositivism research paradigm. This is based on the possibility for having multiple explanations to occurrence of a complex social phenomenon and that objectivity of a researcher may be affected by their own beliefs and values [13].

### 2.1. Data Collection

Participants were intentionally selected for the qualitative interviews and focus group discussions (FGDs) in order to obtain information from different age groups (13–17 years and 18–19 years) and proportionate to the different countries of origin. Other selection criteria of the study participants included having stayed in the refugee camp for at least six months preceding the commencement of the study. After conducting interviews among 28 participants and two FGDs, we obtained saturation with no new emerging information. The interviews were performed mainly in English, Swahili and Kinyarwanda by four field workers who were proficient in these languages. The interviews lasted between 30 to 45 min and the FGDs up to 2 hr. The field workers were trained by the study investigators during a two-day workshop in Mbarara, Uganda. A guide for semistructured interviews and FGDs was developed to guide the interview process, but it also allowed for flexibility. Interviews and FGDs were performed in a private environment, e.g., in the classroom during the break, when other children were outside or in the dormitory room, without a parent/caregiver/teacher being present. During the interviews and FGDs, notes were taken and field workers checked them to ensure accuracy of the records. The questions in the interviews and FGDs were under four broad categories: migration/settlement in Uganda (e.g., “*How long have you been in Uganda? Could you tell us a bit more about your journey*?”); knowledge on menstruation and experiences (e.g., “*How do you understand menstruation*? *Share your menstruation experience.”*—probes first time); menstruation health management (e.g., *“Explain to us how you manage menstruation period.”*—probes availability of hygiene materials, school attendance, challenges); and sources of information or services for menstrual hygiene management (e.g., “*Share with us sources of information or guidance you have received for menstruation management*.”—probes adequacy, sources parents, teachers, peers or media, health facilities, most preferred source).

### 2.2. Data Analysis

The interviews and FGDs were recorded with voice recorders and transcribed. Transcripts were translated into English, when applicable. Data were coded manually using color coding and deductive coding was primarily applied. Two researchers reviewed all the transcripts and generated a codebook and themes based on the literature and qualitative guide. We employed thematic analysis for this research [14]. The steps involved in the analysis correspond with those proposed by Braun and Clarke (2006): become familiar with the data, generate initial codes, search for themes, review themes, define themes and write-up [14]. In order to better illustrate the overarching constructs and themes and the relationships between them, we have consulted the integrated model of menstrual experience suggested by Hennegan et al. (2019) [15]. The topics and subtopics that emerged from the interviews and FGDs are presented in Table 1. The findings are presented with original supporting source quotations from the participants.

### 2.3. Ethical Consideration

The study received approval from the Mbarara University of Science and Technology Research Ethics Committee (REF MUIREC 1/7), the Uganda National Council of Science and Technology (REF SS 4606) and the LMU Ethics Committee, Munich, Germany (Projeckt Nr.: 17-851). A letter of support for the study was obtained from the refugee desk officer and the Nakivale Settlement Commandant. Furthermore, informed consent to participate was directly obtained from adolescents who were 18 years and above, or obtained from parents/guardians, as well as assent, from adolescents below 18 years.

## 3. Results

A total of 28 semistructured interviews and two focus group discussions (FGDs) were performed. Five interviews were excluded from the analysis and results due to low quality, e.g., not clear or very short (Table 2). Girls taking part in the FGDs and interviews represented different countries: Burundi, Rwanda, DR Congo, Ethiopia, Somalia and South Sudan. They fled their home countries due to different conflicts, e.g., South Sudanese Civil War (2013–present), Burundian unrest (2015–2018) and conflicts in DR Congo at different times during 2000–2018.

### 3.1. Social Context for Adolescent Girls in the Nakivale Refugee Settlement

Before describing the experiences of girls around menstruation and its management, we have highlighted some challenges and conditions of living in the settlement, as well as the reasons and circumstances by which girls arrived in Uganda. These can be broadly classified under three main themes, including precarious experiences during migration, family separation and scarcity of resources for livelihood.

#### 3.1.1. Precarious Experiences

Girls shared their stories on how they arrived in Uganda. They were traveling by cars, trucks or walking. However, some girls were very young when their parent(s) had to escape with them to Uganda and they could not recall the circumstances of their travel to Uganda. A number of girls stated that they had to move multiple times across refugee settlements in Uganda as well as across different countries. Risk of violence, lack of money and food insecurity were just few of the multiple challenges girls faced on the way to Uganda. Some girls also reported rape incidents along the way to Uganda with early and unwanted pregnancies as a result:

“I left there [Ethiopia] when it was 2003, when people fought. From Ethiopia we went away to [South] Sudan, then after the fight in 2013 in [South] Sudan, we came here in 2014.”

(16 years old, Ethiopia)

“It was in October 2012; we were running away from the war in Congo. We then lost connections with my sister and we stayed in church there in Congo. In the morning, like at 4:00 am, we went to fetch water. We were like five ladies. We were attacked by thugs and they were many and stronger. They caught all of us and raped us. For me, by bad luck, I became pregnant and that’s how I got my first pregnancy.”

(19 years old, DR Congo)

#### 3.1.2. Family Separation

Not all girls were able to reach Uganda with their families, relatives or caregivers. A few of the girls had to take care of their siblings because they left their home country without parents. Some girls also arrived at the refugee settlement completely alone. A number of adolescent girls were living with either one parent or in foster families:

“I came in September, in 2015. As we were coming from Burundi up to here in Uganda, we got many problems because we didn’t know Uganda. Actually, we didn’t know where we were going, because we were running away from the war and we could not go back. We parted from our parents and lost communication… we had to run and come here. I came with my young siblings [three].”

(19 years old, Burundi)

#### 3.1.3. Scarcity of Resources for Livelihood

Girls mentioned scarcity of basic resources for livelihood including household finances, food supply, scholastic materials, tuition and congestion in living spaces. The majority of participants complained that their living situation was cramped, with tents or rooms shared by many family members, as well as poor hygienic conditions:

“Many people stay in one room. For example, we stay 10 people in one room. For example, we [sisters] stay with our mother in the same room. But my father sleeps in the other room for men.”

(16 years old, South Sudan)

Household finances were described to be dire and food insecurity was a challenge for some families:

“…Also, food, sometimes they [NGOs or The United Nations High Commissioner for Refugees (UNHCR)] give you food for five people when we are like eleven.”

(15 years old, South Sudan)

Schooling is an important issue for the adolescent girls. A number of them mentioned that they were not able to pay school fees and they have received support from local nongovernmental organizations (NGOs), relatives or family members, who stayed in the country of origin, or earned money by doing some small jobs:

“I was given half sponsorship from the Windle Trust [NGO]. I also work for myself during the holidays and I pay my school fees by myself.”

(17 years old, Burundi)

### 3.2. Menstruation and Its Management

The main themes and subthemes from the interviews and FGDs were placed under two overarching constructs: menstrual experience and menstrual management.

#### 3.2.1. Menstrual Experiences

Adolescent girls reported varied experiences regarding menstruation that included being unprepared for menarche, receiving social support on how to handle menstruation and menstrual hygiene, limited exercise or physical activities, school absences, psychological distress and physical pain.

##### First Menstrual Experience

Girls indicated a lack of knowledge about menstruation and its management prior to menarche. Some girls reported myths regarding the cause of bleeding:

“It [menstruation] was alike a miracle to me because I did not know anything about it.”

(18 year old, Rwanda)

“When I was going to the latrine to ease myself, I found blood and after…I came and told her [older cousin] that something has bitten my buttocks. Then she said “no it’s not, it is the one”. Then I asked: “which one”? [smiles].

(16 years old, Ethiopia)

##### Social Support from Family, Siblings, Peers and Teachers

Social support, or lack thereof, strongly dictated menstrual experiences. Parents, siblings, peers, partners and teachers were sources of information, resources, comfort or assistance to accomplish menstrual tasks. Menstruation was positioned as an uncomfortable topic, with little discussion taking place between mother and daughter or between siblings before the first experience. However, girls had mixed experiences during their first time:

“I live with my mum, but I feared to tell her.”

(FGD, 18–19 years old)

“My first time I saw little drops of blood. I was scared and I shared with my mother. She then explained to me everything and asked me to let her know in case the blood continues to come, so that she provides for me what to use.”

(19 years old, Burundi)

“I immediately went to the teacher and told her about the problems that I got from class. Also, my friends in class had to help me with a jacket, because blood passed through my uniform. After going to the teacher, she gave me a pad and asked me to put it on. And when I went home, I told my mother who explained to me more.”

(17 years old, Somalia)

##### Limited Leisure, Exercise and Self-Isolation

There were reports on how menstruation limited the adolescent girls’ ability to participate in leisure or play activity. This was mainly due to pain or feeling unwell. Sometimes they were advised by parents or peers not to engage in physical activities. In some cases, because they had inappropriate hygiene materials, they preferred to self-isolate in fear of staining clothes or embarrassment.

##### School Absences

Several adolescents reported missing schools on some days or the entire duration of menstruation. The fear of having a menstrual accident and subsequent humiliation from peers was also reported to affect school attendance. Girls also reported a feeling of shame and a probability of staining related to the menstruation, which resulted in school absenteeism:

“I did not even have what to use… that made me fear to go to school so that my friends may not laugh at me in case blood leaks out of the dress.”

(19 years old, DR Congo)

“All the days I have it, I don’t come to school. I fear it [the stain] to touch on my clothes that’s why I remain at home.”

(15 years old, Somalia)

##### Psychological Effects

Girls reported various psychological effects related to menstruation including shock, fear, shame and embarrassment. For many of them it was an unexpected surprise or shock to experience it for the first time:

“I was shocked because I didn’t know that I will get menstruation period in that year. I was not old enough.”

(18 years old, Somalia)

“I was scared of leaking because I was using torn clothes.”

(19 years old, Burundi)

##### Physical Experiences

Girls reported experiencing pain that limited movements, activity or even school attendance. A few reported scarcities of pain relief options and not being aware of where to seek help. Some experienced abnormalities of menstruation that required medical attention, such as prolonged bleeding. One respondent reported discomfort during menstruation due to a previous female genital mutilation procedure:

“I actually spent two weeks in menstruation periods. My friends told me that it was a problem, they have advised me to go to the nurse. I went and asked her…”

(17 years old, Burundi)

“On menstrual dates I reach a point when I cannot even lower my foot down…the legs shake… when I step down, I feel a lot of pain. This makes me miss school.”

(FGD, 13–17 years old)

“I sometimes miss exams because of periods, because as you know, I have already told you, that we in Somali culture, we do circumcise girls… And when they circumcise you, they leave a small space there for just urine…”

(18 years old, Somalia)

#### 3.2.2. Menstrual Hygiene Management (MHM) and Practices

Girls reported challenges they faced during menstruation in terms of availability of menstrual and hygiene products, access to health services and advice.

##### Shortage of MHM Supplies

Participants reported the use of disposable menstrual hygiene products when available. These products were obtained from UNHCR that distributed 5–6 packets of pads per woman for an average of six months, which sometimes even had to be shared with other female family members. In absence of these supplies, alternatives reusable materials had to be prepared for use. Most of the other times, they used cloths and rags. However, it was seen that girls were to some extent aware about the importance of proper cleanliness before reusing the products:

“I get a big piece of cloth and cut it into small pieces but not very small. And then I use it in the morning, at eleven I remove it, wash it and hung on the sunshine. In a day I can change like three times. That’s the way how I use those clothes.”

(17 years old, Burundi)

##### Body Hygiene Practices

Several girls reported good hygiene practices during menstruation, including body hygiene and cleaning of reusable materials. The latter were washed with water and soap and sundried:

“I wash with soap and water. And after washing I put it on the rope, after getting dry, I get it and put it on again.”

(14 years old, Ethiopia)

On the other hand, insufficient sanitization and disinfection of the menstrual products were found to threaten the physical health of the participants. They feared the risk of infection in absence of proper menstrual hygiene products:

“As you know when we are in the menstruation periods and use dirty things, there we can get more diseases.”

(19 years old, DR Congo)

##### Seeking Help: Health Care, Family, Friends and Teachers

Female adolescents were comfortable sharing menstruation topics with their friends. However, this was not always possible since the peers of the same age were sometimes unaware. Having older peers with menarche experience was seen to be helpful:

“I asked my friend who was in primary seven, I asked her what to do and she referred me to the school nurse. The school nurse gave me pads and a new knicker.”

(17 years old, Rwanda)

Some of the girls mentioned their teachers as a source of information and assistance:

“My teachers taught me that when you are going to experience it, you go to the toilet and find when all the urine is full of blood.”

(15 years old, South Sudan)

Many of the participants shared that they were unaware of the presence of a health center in the settlement. Others, who were aware, visited the health center for other health problems but not to seek medical help for their menstrual problems. Moreover, adolescents had mixed experiences about the availability of pain killers during menstruation:

“Even now when I feel pain I do not go to the nurse because today I will have the medicine and tomorrow, I will not have it…”

(FGD, 18–19 years old)

“I normally go to Kashozi hospital. It’s where I normally go and get my Panadol [Paracetamol] and they do give us.”

(18 years old, Somalia)

“No! Because I didn’t know where to go. But if the health center was here I would have gone there then.”

(18 years old, Burundi)

## 4. Discussion

This study aimed to describe menstrual health practices and experiences among adolescents in a refugee setting. Here we discuss three key findings of the research, including knowledge and experiences related to menstruation, effect of poor menstrual management on school attendance and social support systems for adolescents to manage menstruation challenges.

This research elaborated on the challenges experienced by the study participants on the way to Uganda. Furthermore, poor living conditions in the settlement, food insecurities and economic hardships to continue schooling were reported. The interplay of these prevailing contextual factors plays a key role in management of menstruation, for instance, having appropriate menstrual hygiene materials or a social support system for addressing key challenges in menstruation.

Regarding menstruation experiences, the study identified lack of knowledge about menstruation and its management prior to menarche among the adolescents. This may lead to anxiety or fear and lack of preparedness on how to handle menstruation. Studies in other low- and lower-middle income countries (LMICs) have reported limited pubertal knowledge prior to onset of menarche [3,16]. The topic of menstruation was often not discussed at home, although some of the participants reported having obtained some information from their teachers. Nevertheless, for most of the participants, first menstruation often came as a surprise and they were unaware of what was happening. Another similar study also reported lack of education on menstrual hygiene management and limited availability of hygiene products among female adolescents in humanitarian emergencies [7].

Lack of proper hygiene products, in addition to problems like excess pain and heavy bleeding during menstruation, impacted schooling, physical health and social participation of the girls.

We noted in this study that poor management of menstruation or menstrual hygiene has a negative impact on school attendance; this has been previously described in related studies among adolescents in low-resource countries [17,18,19]. Adolescent girls in a refugee setting offer a unique context and are more likely to have scarcity of essential needs, including menstrual hygiene materials, access to health facilities, reduced opportunities for self-sustenance and dependence on aid from NGOs. Participants of this study reported an insufficient supply of menstrual hygiene products in the settlement. Economic hardships further made the purchase of these products challenging. Thus, the adolescents were forced to use old cloths and rags, which they washed and dried before reuse. This depicts that they are, to some extent, aware about the risk of infection and the importance of hygiene. However, the implication of sufficient disinfection measures by the adolescents and the availability of the disinfection products are still questionable. Lack of proper hygiene products is indeed problematic and affects not only school attendance but also emotional well-being.

Previous studies in humanitarian settings demonstrated experiences of female adolescents like embarrassment and fear of being teased during menstruation [9,10,11]. Similar experiences were shared by the participants of this study. In order to avoid embarrassment due to leaking, the adolescents stayed away from school and limited their social participation. A previous quantitative publication from the same refugee settlement reported that 43% of the adolescents missed schools during menstruation [12]. Other important factors leading to the school absenteeism were menstrual problems like excessive pain and heavy bleeding. The participants reported excess pain and heavy bleeding during menstruation which impacted schooling, physical health and social participation of the girls. One possible explanation to cyclic periodic pain could be endometriosis and this could not be excluded, particularly in settings where diagnostic facilities are limited [20]. The health seeking behavior of adolescents for menstruation challenges was less than desirable. Participants rarely sought medical help for these problems but rather stayed home and missed school during their monthly cycle. Many of the adolescents were unaware about the presence of a health center in the settlement. Those adolescents visiting the medical facility reported insufficient medical supplies in the health center. In fact, a limited number of adolescents visited the health center to seek advice for their menstrual problems, while most of their visits were for other health conditions, e.g., malaria. Furthermore, experiences of the effects of circumcision during menstruation were also reported during the study suggesting the need of medical assistance for those affected in the settlement. Encouragement to the adolescents to visit the health center for reproductive health issues and provisions for sufficient medical staffs and medical supplies remains crucial.

Psychological effects during menstruation were also reported, thus demonstrating the need for counseling and proper education of the adolescents about the physiology behind menstruation, including the hormonal changes that affect their moods. A qualitative review on menstrual experiences also previously concluded the importance of promoting both physical and psychological health of adolescents [15].

On the other hand, we noted best practices within the refugee community to support adolescents during menstruation. These include support from parents, older peers, and teachers and to some extent health personnel. This kind of support varied from open discussions about hygiene management, supply of materials and pain management. These linkages could be strengthened. UNHCR supplies MHM materials such as pads, although the supplies are often insufficient. There were shared experiences about the use of reusable napkins and maintenance of good hygiene. Promoting support structures for adolescent MHM that could include conducive school environment and training about puberty and MHM, promoting parent adolescent communication on MHM, adolescent health promotion through pain management and other MH complications, as well as support to the girls to make effective reusable napkins could be helpful. Multicomponent and multisectoral engagement to support MHM programming has been found effective [21,22].

### Limitations

This study was qualitative in nature; thus, the objective was not to generate findings that are representative of and generalizable to all the adolescent girls living in the refugee settlements in Uganda, but rather to learn insights about the challenges and needs they face. A convenient sampling method was incorporated, inviting girls from the cross-sectional quantitative survey to participate, which might lead to selection bias and participation of the most motivated and socially engaged girls.

## 5. Conclusions

To ensure a healthy and enjoyable adolescence, it is crucial to provide girls access to timely and evidence-based information and services. Sexuality education and parental involvement play an important role in this process. Furthermore, arrangements for enough health care providers and medical supplies, including psychological counseling, are essential.

## Figures and Tables

**Table 1:** Topics and subtopics that emerged from the data collected during interviews and focus group discussions (FGDs).

Categories	Topics	Subtopics
Social context for adolescent girls in the Nakivale refugee settlement	Precarious experiences	
	Family separation	
	Scarcity of resources for livelihood	
Menstruation and its management	Menstrual experiences	First menstrual experience
		Social support from family, siblings, peers and teachers
		Limited leisure, exercise and self-isolation
		Psychological effect
		Physical experiences
	Menstrual hygiene management (MHM) and practices	Shortage of MHM supplies
		Body hygiene practices
		Seeking help: healthcare, family, friends and teachers

**Table 2:** Age and number of participants in 23 interviews and 2 FGDs included in the study.

Age of the Participants	Number of Participants	Type of the Qualitative Method Used
13–17 years old girls	9	1 FGD
	16	16 Semistructured interviews
18–19 years old girls	10	1 FGD
	7	7 Semistructured interviews
Total number of participants	42	
